# Counselling for Alcohol Problems (CAP), a lay counsellor-delivered brief psychological treatment for harmful drinking in men, in primary care in India: a randomised controlled trial

**DOI:** 10.1016/S0140-6736(16)31590-2

**Published:** 2017-01-14

**Authors:** Abhijit Nadkarni, Benedict Weobong, Helen A Weiss, Jim McCambridge, Bhargav Bhat, Basavaraj Katti, Pratima Murthy, Michael King, David McDaid, A-La Park, G Terence Wilson, Betty Kirkwood, Christopher G Fairburn, Richard Velleman, Vikram Patel

**Affiliations:** a Sangath Centre, Socorro Village, Bardez-Goa, Goa, India; b London School of Hygiene & Tropical Medicine, London, UK; c Department of Health Sciences, University of York, York, UK; d National Institute of Mental Health and Neurosciences, Bengaluru, India; e Division of Psychiatry, University College London, London, UK; f Personal Social Services Research Unit, London School of Economics and Political Science, London, UK; g Department of Psychology, Rutgers School of Arts and Sciences, NJ, USA; h Department of Psychiatry, University of Oxford, Oxford, UK; i Department of Psychology, University of Bath, Bath, UK

## Abstract

**Background:**

Although structured psychological treatments are recommended as first-line interventions for harmful drinking, only a small fraction of people globally receive these treatments because of poor access in routine primary care. We assessed the effectiveness and cost-effectiveness of Counselling for Alcohol Problems (CAP), a brief psychological treatment delivered by lay counsellors to patients with harmful drinking attending routine primary health-care settings.

**Methods:**

In this randomised controlled trial, we recruited male harmful drinkers defined by an Alcohol Use Disorders Identification Test (AUDIT) score of 12–19 who were aged 18–65 years from ten primary health centres in Goa, India. We excluded patients who needed emergency medical treatment or inpatient admission, who were unable to communicate clearly, and who were intoxicated at the time of screening. Participants were randomly allocated (1:1) by trained health assistants based at the primary health centres to enhanced usual care (EUC) alone or EUC combined with CAP, in randomly sized blocks of four to six, stratified by primary health centre, and allocation was concealed with use of sequential numbered opaque envelopes. Physicians providing EUC and those assessing outcomes were masked. Primary outcomes were remission (AUDIT score of <8) and mean daily alcohol consumed in the past 14 days, at 3 months. Secondary outcomes were the effect of drinking, disability score, days unable to work, suicide attempts, intimate partner violence, and resource use and costs of illness. Analyses were on an intention-to-treat basis. We used logistic regression analysis for remission and zero-inflated negative binomial regression analysis for alcohol consumption. We assessed serious adverse events in the per-protocol population. This trial is registered with the ISCRTN registry, number ISRCTN76465238.

**Findings:**

Between Oct 28, 2013, and July 29, 2015, we enrolled and randomly allocated 377 participants (188 [50%] to the EUC plus CAP group and 190 [50%] to the EUC alone group [one of whom was subsequently excluded because of a protocol violation]), of whom 336 (89%) completed the 3 month primary outcome assessment (164 [87%] in the EUC plus CAP group and 172 [91%] in the EUC alone group). The proportion with remission (59 [36%] of 164 in the EUC plus CAP group *vs* 44 [26%] of 172 in the EUC alone group; adjusted prevalence ratio 1·50 [95% CI 1·09–2·07]; p=0·01) and the proportion abstinent in the past 14 days (68 [42%] *vs* 31 [18%]; adjusted odds ratio 3·00 [1·76–5·13]; p<0·0001) were significantly higher in the EUC plus CAP group than in the EUC alone group, but we noted no effect on mean daily alcohol consumed in the past 14 days among those who reported drinking in this period (37·0 g [SD 44·2] *vs* 31·0 g [27·8]; count ratio 1·08 [0·79–1·49]; p=0·62). We noted an effect on the percentage of days abstinent in the past 14 days (adjusted mean difference [AMD] 16·0% [8·1–24·1]; p<0·0001), but no effect on the percentage of days of heavy drinking (AMD −0·4% [–5·7 to 4·9]; p=0·88), the effect of drinking (Short Inventory of Problems score AMD–0·03 [–1·93 to 1·86]; p=0.97), disability score (WHO Disability Assessment Schedule score AMD 0·62 [–0·62 to 1·87]; p=0·32), days unable to work (no days unable to work adjusted odds ratio 1·02 [0·61–1·69]; p=0.95), suicide attempts (adjusted prevalence ratio 1·8 [–2·4 to 6·0]; p=0·25), and intimate partner violence (adjusted prevalence ratio 3·0 [–10·4 to 4·4]; p=0·57). The incremental cost per additional remission was $217 (95% CI 50–1073), with an 85% chance of being cost-effective in the study setting. We noted no significant difference in the number of serious adverse events between the two groups (six [4%] in the EUC plus CAP group *vs* 13 [8%] in the EUC alone group; p=0·11).

**Interpretation:**

CAP delivered by lay counsellors plus EUC was better than EUC alone was for harmful drinkers in routine primary health-care settings, and might be cost-effective. CAP could be a key strategy to reduce the treatment gap for alcohol use disorders, one of the leading causes of the global burden among men worldwide.

**Funding:**

Wellcome Trust.

## Introduction

Alcohol use disorders comprise various conditions related to excessive alcohol consumption, with hazardous drinking, harmful drinking, and dependent drinking reflecting progressively more serious forms.^1^ Alcohol use disorders contribute substantially to disability and premature mortality, accounting for 7·9% (95% CI 6–10) of years lost to disability and 44·4% (29·1–60·0) of years of life lost because of all mental and substance use disorders.^2^ Among men in middle-income countries, alcohol use disorders are the leading neuropsychiatric cause of disease burden.^3^ In India, alcohol-attributable mortality and prevalence of alcohol use disorders relative to the per-person volume of alcohol consumed are high.^1^

**Research in context**

**Evidence before this study**

We updated WHO's Mental Health Gap Action Programme systematic review with our own systematic review. We searched the PubMed, PsycINFO, and IndMed databases from Jan 1, 1990, to Jan 1, 2011, for English language publications using the following search terms: “alcohol”, “drinking”, “addiction”, “psychological”, “therapy”, “counselling”, and “treatment”. Brief psychological treatments based on motivational enhancement have been shown to be effective for management of harmful drinking and are recommended as first-line interventions by WHO's Mental Health Gap Action Programme for delivery in routine health-care settings. However, the existing evidence has low generalisability to many low-income and middle-income countries where both supply side barriers (low availability of mental health professionals) and demand side barriers (low levels of mental health literacy) lead to large treatment gaps.

**Added value of this study**

This study reports the first findings from any low-income and middle-income country assessing the effectiveness and cost-effectiveness of a brief psychological treatment for harmful drinking, delivered by lay counsellors in primary care. The brief (up to four-session) psychological treatment (Counselling for Alcohol Problems), based on motivational enhancement, with additional behavioural and cognitive elements, was better than was enhanced usual care according to all prespecified primary clinical outcomes, except for mean daily alcohol consumed in the past 14 days among those who reported drinking in this period, but no effect occurred on social and functional outcomes. The treatment was readily accepted by this previously untreated population and was highly likely to be cost-effective in this setting.

**Implications of all the available evidence**

Brief psychological treatments for harmful drinking, based on motivational enhancement, are acceptable, feasible, and cost-effective, even when delivered by non-specialist health workers in routine health-care settings in previously untreated populations. Such treatments should be scaled up as one of the key strategies to address the large and rising global burden of alcohol use disorders.

Hazardous (a quantity or pattern of alcohol consumption that places individuals at risk of physical or psychological harm) and harmful (a quantity or pattern of alcohol consumption that has resulted in physical or psychological harm) drinking^4^ affect more people than does dependent drinking^5^ (a quantity or pattern of alcohol consumption characterised by craving, tolerance, a preoccupation with alcohol, and continued drinking despite harmful consequences),^6^ but the policy response to the growing public health problem of alcohol use disorders in low-income and middle-income countries remains focused on dependent drinking.^5^

Various psychosocial interventions are available for treatment of alcohol use disorders and can be broadly summarised as follows. Brief interventions are short, typically a single session lasting up to 15 min, focused on psychosocial interventions designed to address alcohol-related problems or reduce heavy drinking in hazardous drinkers.^7^ Severe alcohol problems, such as harmful drinking, require specialised brief or extended therapies (eg, behavioural therapy, motivational enhancement therapy, or Twelve Step Facilitation).^8^ Although brief psychological interventions are recommended for harmful drinking by the recent Disease Control Priorities Project^9^ (a project aimed at compilation and dissemination of the most up-to-date evidence for cost-effective interventions and their delivery for the leading causes of global disease burden), most people in low-income and middle-income countries, including India, lack access to such interventions because of the absence of skilled human resources^10^ and concerns regarding the contextual appropriateness and generalisability of interventions developed in high-income cultural settings.^11^, ^12^ These barriers could be addressed by development and testing of interventions that have been matched to the context in which they will be offered and delivery of them via non-specialist health workers (NSHWs) or counsellors.^13^

PREMIUM (Program for Effective Mental Health Interventions in Under-Resourced Health Systems) is a research programme whose goal was to design methods for development and assessment of scalable psychological treatments that are culturally appropriate, affordable, and feasible for delivery by NSHWs and to apply these methods^14^ to depression (the Healthy Activity Program [HAP])^15^ and harmful drinking (Counselling for Alcohol Problems [CAP]).^16^ In this Article, we describe the results of a trial assessing the effectiveness and cost-effectiveness of the CAP treatment when used in primary care. The study of HAP treatment is reported separately.^17^ The two trials of HAP and CAP were done concurrently in the same primary health centres (PHCs) and over the same period of time, with the same counsellors delivering both treatments according to the trial allocations of participants.

## Methods

### Study design and participants

In this randomised controlled trial, we recruited participants from PHCs in Goa, India. Of the 14 PHCs in the north district of Goa, the Directorate of Health Services gave permission for PREMIUM to operate in ten. We started screening in eight, but during the course of the trial, two of these PHCs were replaced as one had low attendance and the other had a large proportion of migrant labourers. So at any given time, screening was only happening in eight PHCs. The publicly funded PHCs are the first option for people seeking health care in the public system in India. The population served generally belongs to low socioeconomic groups.

Participants were 18–65-year-old men (women were not eligible as prevalence of any drinking in women in India is low, at 1%^18^) who were likely to be harmful drinkers, defined as scoring 12–19 on the Alcohol Use Disorders Identification Test (AUDIT).^19^ We also included harmful drinkers who screened positive for depression according to the Patient Health Questionnaire 9 (PHQ-9) in this trial; we offered HAP treatment to those who continued to screen positive for depression at the end of CAP treatment. Although we did offer people with alcohol dependence (ie, those who scored higher than 20) the opportunity to participate in the trial, this action was taken primarily to enhance the acceptability of the programme in the PHCs; as the trial was not powered for outcomes in this opportunistically identified group, the findings are not reported here. We excluded from screening patients who needed emergency medical treatment or inpatient admission, who were unable to communicate clearly, and who were intoxicated at the time of screening.

The trial protocol^20^ was approved by the Trial Steering Committee, and ethical approval for the conduct of the trial was obtained from the Institutional Review Boards of the London School of Hygiene & Tropical Medicine, Sangath (the implementing institution in India), and the Indian Council of Medical Research. Written (or witnessed, if the participant is illiterate) informed consent was mandatory for enrolment. We audiotaped all consent procedures, with patients' approvals.

### Randomisation and masking

A randomisation list in randomly sized blocks (four to six), stratified by PHC, was generated by a statistician independent of the trial. The randomisation code was concealed and allocated by trained health assistants based at the primary health centres at the individual level after completion of the baseline assessments using sequential numbered opaque sealed envelopes.^21^ Physicians providing enhanced usual care (EUC) were masked to allocation status, as were the independent assessors who did the outcome assessments, and these people had no contact with the PHCs or other team members. All authors, apart from the data manager (BB), were masked until the trial results were unmasked. Instances of unmasking of outcome assessors in the CAP group will be summarised on the basis of overall prevalence and the exact point during the interview that the interviewer was unmasked.

### Procedures

Trained health assistants, independent of the counsellors, screened patients using AUDIT and administered a baseline questionnaire to trial participants to collect sociodemographic information (eg age and marital status) and data for potential moderators of treatment effect. We audiotaped all outcome interviews (with permission), and the tapes were randomly selected (using a random selection strategy stratified by outcome assessor) for review by the supervisor for quality assurance.

In the EUC group, usual care (consultation with the PHC physician) was enhanced by provision of the screening results to the PHC physician and provision of a contextualised version of the WHO Mental Health Gap Action Programme guidelines^22^ for harmful drinking, including when and where to refer patients for specialist care. In the CAP group, participants received EUC plus CAP. CAP is a manualised psychological treatment delivered in three phases over a maximum of four sessions (each lasting approximately 30–45 min) at weekly to fortnightly intervals.^15^ The initial phase involves detailed assessment followed by personalised feedback; the middle phase involves helping the patient to develop cognitive and behavioural skills and techniques, consisting of drink refusal skills, handling of peer pressure, problem-solving skills, and handling of difficult emotions; and the ending phase involves the patient learning how to manage potential or actual relapses using the skills acquired in the middle phase.

The stance adopted by the counsellor is that of motivational interviewing^23^ and client-centred general counselling strategies (eg, open-ended questioning and showing of empathy). The general counselling and problem-solving strategies were shared between CAP and HAP treatments. We typically conducted sessions face-to-face, at the PHC or patient's home, but used telephone sessions when necessary. We considered patients who missed three consecutive scheduled sessions to have dropped out of treatment. Counsellors were adults with no professional training or qualification in the field of mental health, they had completed at least secondary school education, and they were fluent in the vernacular languages used in the study settings. The selection process began with interviews involving roleplays for applicants for the training; for those who cleared this step, the process continued with intensive 2 week classroom training in both CAP and HAP treatments, followed by a competency assessment; for those who graduated this step, the process continued with a 6 month internship with supervision of cases by experts; and finally, selection occurred through testing of knowledge (multiple choice question exam) and skills (roleplays with use of standardised vignettes and quality ratings of actual CAP sessions delivered). 11 counsellors participated in the trial. They received weekly peer-led supervision in groups of four to six, which involved rating of a randomly selected (using a random selection strategy stratified by counsellor and phase of session) 10% of recorded sessions on the CAP Therapy Quality Scale (TQS)^24^ and individual supervision twice monthly. We used information about contact with the counsellor to estimate CAP delivery costs, which took into account training, supervision, and salary costs.

We assessed treatment fidelity via treatment completion, maintained by counsellors in their clinical records, CAP TQS scores from peer and expert ratings of audio recordings of sessions during weekly group supervision,^24^ and therapy quality of a random selection (using a random selection strategy stratified by counsellor and phase of session) of 10% of all sessions by an expert involved in the development of CAP.

### Outcomes

Primary outcomes were remission defined as an AUDIT score of less than 8 and mean daily alcohol consumption in the past 14 days immediately preceding the 3 month outcome assessment. We measured the primary outcomes 3 months after enrolment. Secondary outcomes were the Short Inventory of Problems (SIP) mean score, WHO Disability Assessment Schedule (WHODAS) II mean disability score, total days unable to work in the previous month, a suicide attempt in the past 3 months, perpetration of intimate partner violence (“In the past 3 months, have you slapped, hit, kicked, punched your wife/partner or done something else that did or could have hurt her physically?”), and resource use and costs of illness estimated from the Client Service Receipt Inventory.^25^ SIP mean score was prespecified as a primary outcome in the protocol; however, in a joint meeting of the Trial Steering Committee and Data Monitoring and Safety Committee before unmasking, SIP mean score was changed to a secondary outcome to reduce multiplicity of the primary outcomes. two additional secondary outcomes that were not prespecified (percentage of days abstinent and percentage of days of heavy drinking generated from the Timeline Followback) were also added to bring the trial in line with recommendations of the National Institute on Alcohol Abuse and Alcoholism.^26^ We did outcome assessment between Jan 29, 2014, and Nov 30, 2015. We collected data for serious adverse events, defined as deaths, suicide attempts, and unplanned admissions to hospital from any cause.

### Statistical analysis

Based on the assumptions of participants being randomly allocated within each of the clinics, of there being one counsellor per PHC at any one time, of an intracluster correlation of 0·04, of a loss to follow-up of 15% over 3 months, and of a 1:1 allocation ratio, a trial size of 400 enrolled participants with harmful drinking had 90% power to detect the hypothesised effects (effect size of 0·45 for mean standard ethanol content consumed; remissions of 68% *vs* 40% in favour of CAP) for the primary outcomes, with a 5% type I error. In estimating the sample size, we considered both primary outcomes, and the study was adequately powered to assess each of these outcomes independently. For the binary primary outcome of remission, we had a 99% power to detect a remission of 68% in the EUC plus CAP group versus 40% in the EUC alone group, and for the continuous primary outcome of daily alcohol consumption, we had a 93% power to detect an effect size of 0·45.

Analyses were on an intention-to-treat basis, with multiple imputation for missing outcome data assuming data were missing at random, assuming predictive mean matching for positively skewed outcomes. We assessed serious adverse events in the per-protocol population. We estimated the primary continuous outcome (mean daily ethanol consumed in the past 14 days) by multiplying the total standard drinks^27^ consumed in the past 14 days by 10 (based on the WHO definition of a standard drink as 10 g of pure ethanol^28^). We used zero-inflated negative binomial regression^29^ to estimate the intervention effect for this outcome and other positively skewed overdispersed outcomes with an excess of zeros. We analysed continuous outcomes with normally distributed residuals using linear regression. We analysed binary outcomes using logistic regression.

We adjusted all models for both PHC as a fixed effect to allow for within-PHC clustering and for baseline AUDIT score. As only ten PHCs were included in the study, we decided to adjust for these PHCs as fixed effects, as recommended by Kahan^30^ for studies with a small number of centres. However, we did a sensitivity analysis using random-effects models to adjust for within-PHC clustering. Additionally, we did a post-hoc analysis allowing for clustered errors using the cluster option in Stata. For outcomes analysed with use of zero-inflated negative binomial regression, the intervention effect is estimated for all participants in one model as an adjusted odds ratio with a 95% CI for the proportion with zero (ie, no reported drinking) and an adjusted count ratio with a 95% CI among those with non-zero responses. For other continuous outcomes, we reported the intervention effect as the adjusted mean difference with a 95% CI. For binary outcomes, we reported the intervention effect as the adjusted prevalence ratio and adjusted prevalence difference, estimated using the marginal standardisation technique with a 95% CI for the prevalence ratios estimated using the δ method.^31^ We assessed moderators of treatment effect for a-priori-defined moderators, namely baseline severity of drinking, readiness to change, and expectations of the usefulness of counselling. Sensitivity analyses for linear and logistic regression models were adjustment for counsellor as a random effect, and complete case analysis. We describe results in terms of the strength of evidence rather than statistical significance,^32^ and the consistency of results for related outcomes are examined to interpret findings.

We did economic assessments (comparative analysis of costs and outcomes between EUC plus CAP and EUC alone groups) from the health-care system and societal viewpoints. We estimated the costs of CAP by attaching appropriate local Indian unit costs to each resource required to deliver each component of the intervention, including training, supervision, travel, and materials. We also collected detailed information about total counsellor time for all attempted and completed contacts, including travel time, valued using actual counsellor salaries. We used the Client Service Receipt Inventory to record participants' subsequent contacts with health services, including hospital inpatient and outpatient contacts, and also to document any patient-borne or family-borne costs, including time out of their usual occupation. We valued time out of usual occupation for patients and their families using relevant published mean wages.

We compared changes in principal outcomes with changes in costs to calculate Incremental Cost Effectiveness Ratios (ICERs). We calculated cost per additional remission or non-drinker achieved and quality-adjusted life-year (QALY) gained. We compared differences in mean costs using standard parametric tests. We derived QALY scores through transformation of WHODAS II 12 item scores.^33^ We imputed missing values for QALYs and cost data and bootstrapped ICERs to derive 95% CIs. We explored statistical uncertainty around the ICERs through cost-effectiveness acceptability curves showing the likelihood that CAP would be cost-effective at different levels of willingness-to-pay thresholds. All costs are presented in 2015 international dollars. We did statistical analyses using Stata version 14.1. A Data and Safety Monitoring Committee oversaw the trial. This trial is registered with the ISCRTN registry, number ISRCTN76465238.

### Role of the funding source

The funder of the study had no role in study design, data collection, data analysis, data interpretation, or writing of the report. VP, HAW, AN, BW, DM, A-LP, and BB had full access to all the data in the study. VP, AN, and BW had final responsibility for the decision to submit for publication.

## Results

**Figure 1:**
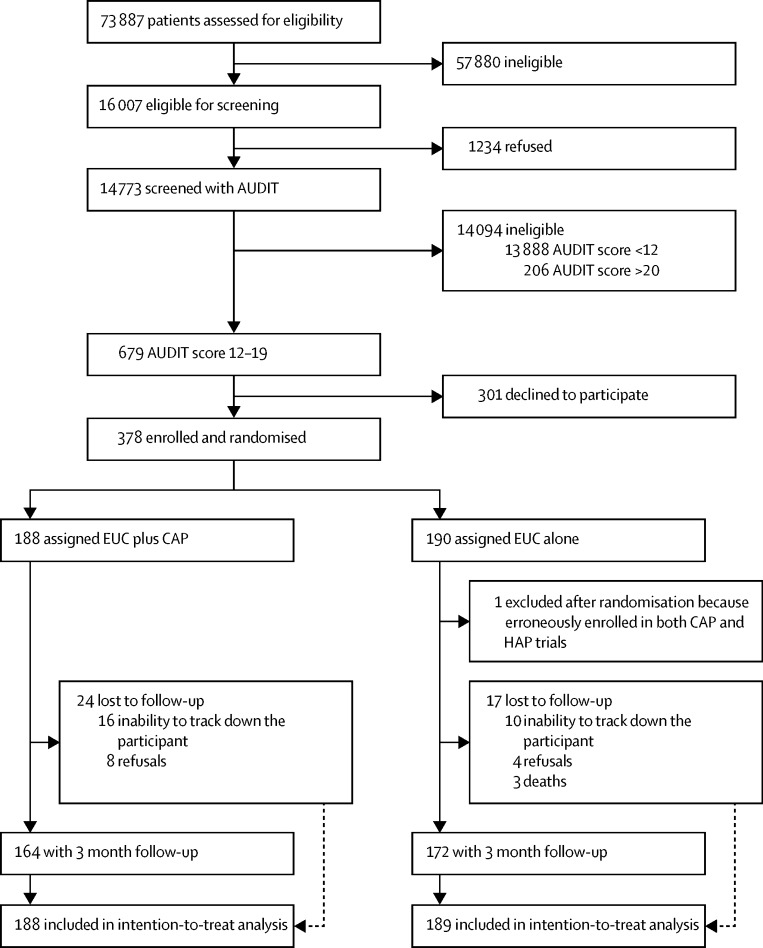
Trial profile AUDIT=Alcohol Use Disorders Identification Test. CAP=Counselling for Alcohol Problems. EUC=enhanced usual care. HAP=Healthy Activity Program.

Between Oct 28, 2013, and July 29, 2015, we assessed 73 887 PHC attenders for eligibility (figure 1). Of these, 16 007 (22%) were eligible for screening and 14 773 (92%) of these were screened with AUDIT. Of these, 679 (5%) screened positive as harmful drinkers and 378 (56%) of these consented to participate and were enrolled and randomly allocated (188 [50%] to the EUC plus CAP group and 190 [50%] to the EUC alone group, one of whom was subsequently excluded from the EUC alone group because he was erroneously enrolled in both CAP and HAP trials, leaving a total of 189 patients in the EUC alone group). The leading reasons for ineligibility for screening included age younger than 18 years or older than 65 years (23 453 [41%] of 57 880), already having been screened within the last 3 months (10 046 [17%]), not planning to be resident in the study area for the duration of the study (9835 [17%]), and being resident outside of the study catchment areas (6014 [10%]). The trial ended on Aug 30, 2016, when the 12 month outcome assessment ended.

Baseline characteristics were similar between groups (table 1). We noted no significant difference in age between participants and those who declined participation (mean age 42·0 years [SD 11·4] *vs* 40·5 years [11·7]; p=0·09), with a higher AUDIT (median AUDIT score 14 [IQR 13–16] *vs* 14 [13–16]; p=0·04) and PHQ-9 (median PHQ-9 score 4 [IQR 1–8] *vs* 3 [1–6]; p<0·0001) score. Participation by PHC varied significantly (p=0·001), with higher participation in some PHCs (>60%) rather than others (<60%; appendix). Of the 377 participants, 336 (89%) were seen at the primary endpoint of 3 months (164 [44%] in the EUC plus CAP group and 172 [46%] in the EUC alone group), a figure similar to the number predicted for the sample size estimation. Participants who were lost to follow-up tended to be younger than were those not lost to follow-up (appendix). Reasons for loss to follow-up were inability to track down the participant (26 [63%] of 41; 16 [39%] in the EUC plus CAP group *vs* ten [24%] in the EUC alone group), refusal (12 [29%]; eight [20%] *vs* four [10%]), and death (three [7%] in the EUC alone group). We imputed outcome data for these 41 participants lost to follow-up.

The proportion with remission according to AUDIT was significantly higher in the EUC plus CAP group than in the EUC alone group (59 [36%] of 164 scoring less than 8 on AUDIT in the EUC plus CAP group *vs* 44 [26%] of 172 in the EUC alone group; adjusted prevalence ratio 1·50 [95% CI 1·09–2·07]; p=0·01; adjusted prevalence difference 12·6% [5·9–27·1]; table 2). Analysis of daily ethanol consumption showed a significantly higher proportion of participants reporting no alcohol consumption in the past 14 days in the EUC plus CAP group than in the EUC alone group (68 [41%] in the EUC plus CAP group *vs* 31 [18%] in the EUC alone group; adjusted odds ratio 3·00 [95% CI 1·76–5·13]; p<0·0001) and no difference in consumption among those who reported any drinking in this period (37·0 g [SD 44·2] *vs* 31·0 g [27·8]; count ratio 1·08 [95% CI 0·79–1·49]; p=0·62).

**Figure 2:**
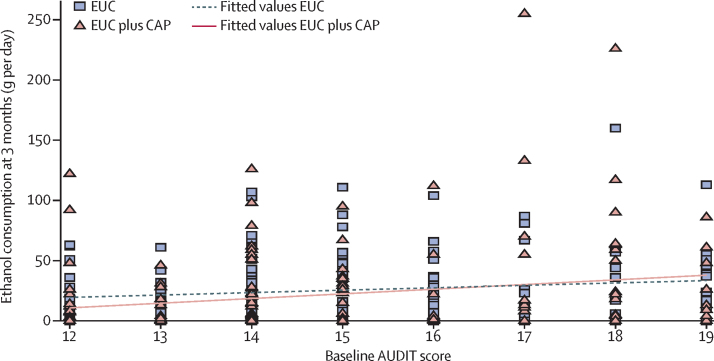
Ethanol consumption at 3 months by baseline AUDIT score AUDIT=Alcohol Use Disorders Identification Test.

We noted no evidence of an intervention effect on SIP score, WHODAS II score, days unable to work, suicide attempts, perpetration of intimate partner violence, and percentage of days of heavy drinking. We did note a significant intervention effect on the percentage of days abstinent in the past 14 days. We noted no evidence of effect modification by baseline AUDIT score (figure 2) or expectations of the usefulness of counselling (appendix). However, we found evidence of a greater intervention effect among those not already trying to change drinking behaviour at baseline for ethanol consumption (p=0·003) than among those already trying. Results were similar when adjusted for counsellor as a random effect, when using complete case analyses, and when allowing for clustered errors. We noted no significant differences in the number of serious adverse events between the two groups (any serious adverse event six [4%] in the EUC plus CAP group *vs* 13 [8%] in the EUC alone group, p=0·11; death none *vs* three [2%], p=0·25; suicide attempts none *vs* three [1%], p=0·25; unplanned admissions to hospital six [4%] *vs* seven [4%], p=1·00; appendix).

The intraclass correlation for ethanol consumption at 3 months within PHCs was 0·04, as predicted. Of the 188 participants in the EUC plus CAP group, 131 (70%) had a planned discharge and none were referred for specialist care. The mean number of sessions for those who had a planned discharge was 2·8 (95% CI 2·7–3·0), whereas those who had an unplanned discharge were most likely to drop out after the first session (mean number of sessions 1·1 [95% CI 1·0–1·3]). Of the total of 434 sessions delivered, 425 (98%) were delivered in face-to-face format; 84 (33%) of 257 sessions from the second session onwards were delivered at home, and 42 (22%) of participants in the EUC plus CAP group had a significant other involved in at least one session. The mean duration of sessions was 42·4 min (40·9–43·7). Mean TQS score on the basis of peer supervisor ratings (n=183) was 2·35 (2·29–2·41), similar to expert supervisor ratings (n=183; mean 2·44 [2·36–2·51]) and the mean score of the independent rater for 10% of randomly selected sessions (n=40; mean 2·64 [2·42–2·87]), indicating adequate to good therapy quality. 13 (3%) of 377 investigators were unmasked, with eight (2%) unmasked before the primary outcome assessment.

**Figure 3:**
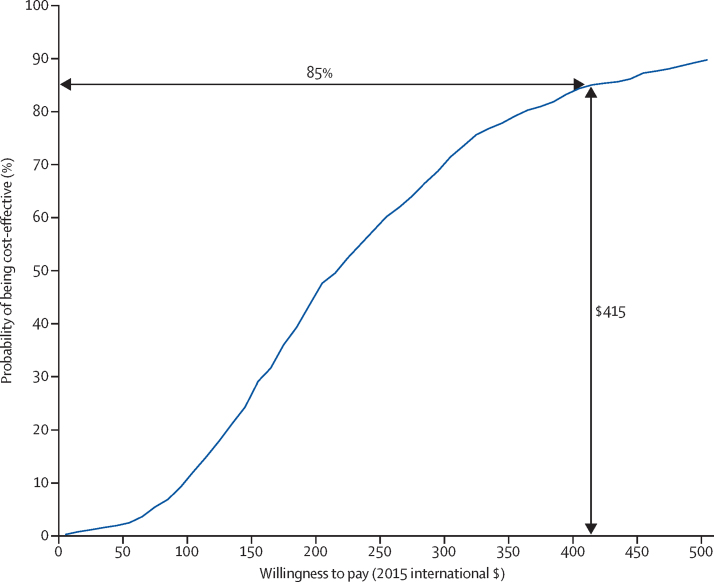
Cost-effectiveness acceptability curve: willingness to pay per remission achieved from Counselling for Alcohol Problems from a health system perspective

From the health system perspective, the total health-care cost per person—ie, including the intervention cost, was significantly higher in the EUC plus CAP group than in the EUC alone group, with no significant difference in QALY scores (table 3). Excluding intervention costs, we noted no significant differences in aggregate health-care costs. Medication costs were significantly lower in the EUC plus CAP group than in the EUC alone group. The incremental cost per additional remission from a health-care system perspective is shown in table 4; as figure 3 shows, if society is willing to pay up to the monthly minimum wage in Goa ($415)^34^ per individual in remission, CAP has an 85% chance of being cost-effective. Similarly, the cost per additional non-drinker was $124 (95% CI −$102 to $325), which would mean that CAP would have a more than 99% chance of being considered cost-effective (appendix).

## Discussion

This study provides evidence of the effectiveness of CAP, a brief psychological treatment for harmful drinking delivered by NSHWs in routine primary care settings. CAP was associated with strong effects on abstinence and remission 3 months after enrolment, but had no effect on other alcohol-related outcomes. The economic analysis indicates that CAP is likely to be cost-effective with regard to remission and non-drinking outcomes.

Although WHO recommends brief counselling for treatment of harmful drinkers,^35^ almost all evidence in support of these recommendations is from high-income settings.^7^ Our results add to the evidence base by showing that multisession brief interventions for harmful drinking in primary care attenders can be effective when delivered by well trained and supervised health workers without any previous mental health training.^36^ Only two randomised controlled trials^37^, ^38^ in low-income or middle-income countries have tested a NSHW-delivered treatment for any form of alcohol use disorder. However, both of these previous studies targeted hazardous or binge drinkers, and only one^37^ was based in primary care. Our study is the first, to our knowledge, of such an intervention that has reported an economic assessment. The CAP treatment is a contextually appropriate intervention for harmful drinking developed specifically to be delivered by lay counsellors in primary care settings in low-income or middle-income countries, and this study provides evidence of effectiveness compared with EUC. When wide societal effects (eg, domestic violence and law enforcement costs) and savings for the family are considered, the economic case could perhaps be further strengthened. Although the economic argument might be a cause for action, it needs to be tested in future assessments.

As described above, brief interventions typically involve a brief conversation delivered to hazardous drinkers. For harmful drinkers, brief therapies focusing on specific behavioural change strategies, including provision of clients with skills to deal with alcohol-related problems, might be appropriate.^39^, ^40^ The CAP treatment seeks to do just that. In India, various alcoholic beverages are consumed, consisting of commercial, licit non-commercial, and illicit home-brewed alcoholic beverages.^1^ The CAP treatment is designed around assessment, personalised feedback, and provision of skills needed to manage behaviours related to drinking, irrespective of the specific type of alcohol consumed. The pattern of outcomes suggests that CAP had effects on those who chose abstinence as a treatment goal, but did not have any effects on those who chose to continue drinking. This finding is consistent with the prevailing beliefs about the nature of alcohol problems in India, which place great importance on abstinence.^41^ The greater effect of CAP on those who were not already trying to make a change in their drinking behaviour compared with those who had already started to make a change indicates that the treatment enhanced motivation to change. This finding is consistent with the motivational enhancement theory on which CAP is based.^42^ CAP did not have any significant effect on the adverse consequences of alcohol use disorder, as shown by the absence of significant differences in any of the prespecified secondary outcomes. One probable reason for this finding is that the severity of harmful drinking is not great enough to register on tools like SIP whose previous use has been primarily for people with dependent drinking, and consequently, any intervention targeting such drinking patterns does not result in observed changes with these tools. Finally, changes in outcomes like perpetration of domestic violence might possibly require specific strategies targeting these behaviours, and only targeting of drinking as a mediating mechanism might not be effective in reduction of domestic violence.

The study had several limitations. Reliance on self-reported outcome data entails susceptibility to social desirability bias, and this factor might have varied by group.^43^ Reasons for under-reporting might have included the participant actually believing the information that they reported (self-deception) or so-called faking good to conform to socially acceptable values, avoid criticism, or gain social approval. However, more objective measures such as biomarkers are insensitive to alcohol use disorder except for when it is severe, and alcohol treatment trials have not found advantages in use of collateral reports or other alternatives.^44^ Biomarkers might, in time, be developed for use in clinical trials, although at present the most promising ones available do not accurately and sensitively estimate levels of consumption.^45^ The results in our study are restricted to the primary outcomes at 3 months where our interest lies in the response and remission of participants with harmful drinking after our treatment. We intend to assess the sustainability of these outcomes, including recovery from harmful drinking, at a 12 month follow-up. No cost-effectiveness thresholds have been established for alcohol outcomes in India; we have conservatively assumed that this threshold is no more than the monthly wage for an unskilled worker. The absence of effect on QALYs might be viewed in the context of doubt about the capacity of standard measures such as those used in this study to capture improvements in alcohol-related quality of life.^46^ A delayed effect of reduced drinking or abstinence on QALYs could also be possible and we could perhaps expect to see a differential effect between the two groups at the 12 month outcome assessment. Finally, our findings cannot be generalised to women as CAP was developed and tested only in men. However, none of the content of CAP is sex specific and in our opinion there is no theoretical reason to believe that CAP would not work in women. Nevertheless, as alcohol consumption and its resulting problems are starting to increase in India, albeit from an extremely small base, study of this treatment in female harmful drinkers is needed.

The strengths of this trial lie in its design and the rigorous procedures followed in its implementation. The ratings of therapy quality, both independent and by supervisors, and the high levels of treatment completion testify to the acceptability and feasibility of this non-specialist-delivered treatment. Another strength was that intensive assessments were not done at baseline as assessment reactivity has been found to be problematic in alcohol use disorder trials.^47^, ^48^ Considered together with the companion study,^17^ the two PREMIUM trials represent a substantial achievement in global mental health for several reasons. First, the interventions are brief, delivered by lay people and provided to primary health-care attenders with few exclusion criteria, thus enhancing their generalisability to routine health care. Second, the treatment was delivered by the same counsellors who concurrently delivered the treatment for depression, mimicking the real world where patients would have a single counsellor in a health facility simultaneously treating the two leading mental health disorders worldwide. Third, the treatments are built around a theoretical orientation, which have a strong grounding in the psychological treatment literature. Finally, the trials report for the first time evidence for the cost-effectiveness of psychological treatments for these two common mental health conditions from a low-income or middle-income country.

Further research should focus on replication and assessment of CAP's effects on severe forms of alcohol use disorder, including as a component of a stepped care intervention for the full range of severity of alcohol use disorder. Our dissemination efforts for CAP include launching of an online platform for those interested to learn about the treatment^49^ and an online documentary about the PREMIUM trials.^50^

## Figures and Tables

**Table 1:** Baseline characteristics

		**EUC plus CAP (n=188)**	**EUC alone (n=189)**
Age (years)		42·3 (11·8)	41·7 (10·9)
Marital status			
	Married	147 (78%)	154 (81%)
	Single	38 (20%)	32 (17%)
	Separated, divorced, or widowed	3 (2%)	3 (2%)
Occupation			
	Unemployed	25 (13%)	28 (15%)
	Unskilled manual labour	131 (70%)	135 (71%)
	Skilled manual labour	13 (7%)	12 (6%)
	Clerical and professional	19 (10%)	14 (7%)
Education			
	No formal education	41 (22%)	29 (15%)
	Completed primary education	90 (48%)	107 (57%)
	Completed secondary education or higher	57 (30%)	53 (28%)
Patient's expectation of usefulness of counselling			
	Not useful	1 (1%)	2 (1%)
	A little or somewhat useful	36 (19%)	39 (21%)
	Moderately useful	42 (22%)	38 (20%)
	Very useful	109 (58%)	110 (58%)
AUDIT score			
	Mean	14·7 (2·1)	15 (2·1)
	Median	14 (13–16)	15 (13–17)

Data are mean (SD), n (%), or median (IQR).

**Table 2:** Primary and secondary outcomes

		EUC plus CAP (n=164)*	EUC alone (n=172)*	**Intervention effect (95% CI)**†	**p value**
**Primary outcomes**					
Remission (AUDIT score of <8)		59 (36%)	44 (26%)	aPR 1·50 (1·09–2·07)	0·01
Daily standard ethanol consumed in the past 14 days‡					
	Non-drinkers	68 (41%)	31 (18%)	aOR 3·00 (1·76–5·13)	<0·0001
	Ethanol consumption among drinkers (g)	37·0 (44·2)	31·0 (27·8)	Count ratio 1·08 (0·79–1·49)	0·62
**Secondary outcomes**					
	SIP score	7·9 (9·1)	8·2 (8·9)	AMD −0·03 (−1·93 to 1·86)	0·97
	WHODAS II score	4·4 (6·2)	3·5 (5·3)	AMD 0·62 (−0·62 to 1·87)	0·32
Days unable to work‡					
	None	109 (66%)	117 (68%)	aOR 1·02 (0·61–1·69)	0·95
	Days unable to work when at least 1 day reported	11·5 (10·4)	11·2 (10·1)	Count ratio 0·92 (0·59–1·43)	0·70
Number of suicide attempts		0	3 (2%)	aOR 0; aPR 1·8 (−2·4 to 6·0)	0·25
Perpetration of intimate partner violence§		12/127 (9%)	16/140 (11%)	aOR 0·81 (0·39–1·67); aPR 3·0 (−10·4 to 4·4)	0·57
Percentage of days abstinent		69·4% (37·3)	54·4% (36·3)	AMD 16·0% (8·1 to 24·1)	<0·0001
Percentage of days of heavy drinking		9·5% (2·5)	10·0% (2·4)	AMD −0·4% (−5·7 to 4·9)	0·88

Data are n (%) or mean (SD). EUC=enhanced usual care. CAP=Counselling for Alcohol Problems. AUDIT=Alcohol Use Disorders Identification Test. aPR=adjusted prevalence ratio. aOR=adjusted odds ratio. SIP=Short Inventory of Problems. AMD=adjusted mean difference. WHODAS=WHO Disability Assessment Schedule.

* Among those with observed data at 3 months.

† Including imputed outcome data for those with missing data.

‡ Analysed with a zero-inflated negative binomial model that fits two parameters in one model—ie, the proportion with response of zero (eg, no drinking in 14 days or no days unable to work) and the mean count (eg, ethanol consumption or days unable to work) among people with a non-zero (positive) response.

§ Among married participants only.

**Table 3:** Costs per person and cost-effectiveness analyses (2015 international dollars)

	**EUC plus CAP (n=188)**	**EUC alone (n=189)**	**Mean difference (95% CI)**	**p value**
**Health system costs ($)**				
PHC doctor consultations	$7 (12)	$9 (5)	−$2 (−5 to 1)	0·11
Hospital doctor consultations	$3 (12)	$3 (9)	−$0 (−2 to 2)	0·77
Hospital admissions	$13 (92)	$13 (56)	$0 (−16 to 14)	0·89
Laboratory tests	$4 (9)	$6 (21)	−$2 (−6 to 0)	0·08
Medicines	$4 (10)	$7 (18)	−$3 (−7 to 1)	0·02
Total public health-care costs	$30 (104)	$38 (76)	−$8 (−26 to 11)	0·40
CAP treatment	$33 (30)	$0	$33 (2 to 38)	<0·0001
**Productivity costs ($)**				
Time costs to service users and families	$23 (47)	$19 (33)	$4 (−6 to 9)	0·80
Productivity losses	$53 (110)	$64 (119)	−$11 (−37 to 9)	0·24
**Total costs ($)**				
Health system perspective	$64 (111)	$39 (77)	$25 (5 to 44)	0·01
Societal perspective	$139 (211)	$121 (169)	$18 (−18 to 59)	0·30
**Cost-effectiveness analyses**				
QALYs gained	0·220 (0·013)	0·221 (0·012)	−0·001 (−0·004 to 0·001)	0·29

Data are mean (SD). EUC=enhanced usual care. CAP=Counselling for Alcohol Problems. PHC=primary health centre. QALY=quality-adjusted life-year.

**Table 4:** Cost-effectiveness analyses from health system and societal perspectives (2015 international dollars)

	**Health system perspective**	**Societal perspective**
Cost per remission ($)	$217 (50 to 1073)	$150 (−216 to 1051)
Cost per non-drinker ($)	$124 (−102 to 325)	$86 (29 to 265)
Cost per QALY gained ($)*	−$17 710 (−220 368 to 141 383)	−$12 267 (−104 070 to 133 648)

Data are mean (95% CI).

* Negative values reflect the lower QALY score and not lower costs.
