# Supplementary material for: Counselling for Alcohol Problems (CAP), a lay counsellor-delivered brief psychological treatment for harmful drinking in men, in primary care in India: a randomised controlled trial
Source: Lancet. 2017 Jan 14;389(10065):186–95. doi: 10.1016/S0140-6736(16)31590-2 (PMC5236065; doi:10.1016/S0140-6736(16)31590-2)

THE LANCET

Supplementary appendix

This appendix formed part of the original submission and has been peer reviewed. We post it as supplied by the authors.

Supplement to: Nadkarni A, Weobong B, Weiss HA, et al. Counselling for Alcohol Problems (CAP), a lay counsellor-delivered brief psychological treatment for harmful drinking in men, in primary care in India: a randomised controlled trial. *Lancet* 2016; published online Dec 14. http://dx.doi.org/10.1016/S0140-6736(16)31590-2.

Appendix

Supplementary Table 1: Sensitivity analyses and effect modification for the 'Remission' outcome

**Analysis**	**EUC + CAP**	**EUC alone**	**Adjusted prevalence ratio (95% CI)**	**p-value**
**Sensitivity analyses**				
Original analysis (MI)			1.50 (1.09, 2.07)	P=0.01
Complete case	59/164 (35.98%)	44/172 (25.58%)	1.44 (1.04, 1.99)	P=0.03
Complete case adjusting for counsellor as random effect	59/164 (35.98%)	44/172 (25.58%)	1.47 (0.97, 1.97)	P=0.08
MI adjusting for counsellor as random effect			1.54 (1.02, 2.05)	P=0.02
**Effect modification**				
**Baseline AUDIT**			p-value for effect modification=0.88	
12-15	40/107 (37.38%)	27/100 (27.00%)	1.41 (0.95, 2.11)	P=0.09
16-19	19/57 (33.33%)	17/72 (23.61%)	1.44 (0.82, 2.53)	P=0.20
**Trying to change**			p-value for effect modification=0.14	
Not already trying	30/78 (38.46%)	17/85 (20.00%)	2.00 (1.19, 3.33)	P=0.008
Already trying	29/86 (33.72%)	27/87 (31.03%)	1.14 (0.74, 1.76)	P=0.54
**Expectations of usefulness of counselling**			p-value for effect modification=0.59	
Not or somewhat useful	11/36 (30.56%)	9/38 (23.68%)	1.43 (0.64, 3.17)	P=0.38
Moderately or very useful	48/128 (37.50%)	35/134 (26.12%)	1.50 (1.05, 2.14)	P=0.02

Supplementary Table 2: Sensitivity analyses and effect modification for the 'ethanol consumption' outcome

**Analysis**	**EUC + CAP**	**EUC alone**	**aOR or count ratio (95% CI)**	**p-value**
**Sensitivity analyses**				
Original analysis (MI)			aOR=3.00 (1.76, 5.13)<br>Count ratio=1.08 (0.79, 1.49)	P<0.0001<br>P=0.62

Complete case	Non drinkers: 68/164 (41.46%)<br>Mean in drinkers: 37.00 (44.21)	31/172 (18.02%)<br>31.05 (27.77%)	aOR=3.58 (2.04, 6.29)<br>Count ratio=1.03 (0.79, 1.36)	P<0.0001<br>P=0.81
Complete case adjusting for counsellor as random effect[1]			aOR=3.58 (2.78, 4.61)<br>Count ratio=1.03 (0.80, 1.34)	P<0.0001<br>P=0.80
MI adjusting for counsellor as random effect			aOR=3.00 (2.16, 4.18)<br>Count ratio=1.08 (0.80, 1.47)	P<0.0001<br>P=0.60
**Effect modification**				
**Baseline AUDIT score**			Overall p-effect modification=0.07[2]<br>p-effect modification (aOR)=0.08<br>p-effect modification (Count ratio)=0.15	
12-15	Non drinkers: 47/107 (43.93%)<br>Mean in drinkers: 29.62 (30.22)	14/100 (14.00%)<br>28.67 (25.11)	aOR=4.51 (1.94, 10.51)<br>Count ratio=0.94 (0.67, 1.31)	P=0.0001<br>P=0.71
16-19	Non drinkers: 21/57 (36.84%)<br>Mean in drinkers: 49.31 (59.29)	17/72 (23.61%)<br>34.76 (31.36)	aOR=1.91 (0.84, 4.38)<br>Count ratio=1.19 (0.74, 1.91)	P=0.12<br>P=0.47
**Trying to change**			**Overall p-effect modification=0.003**[2]<br>p-effect modification (aOR)=0.006<br>p-effect modification (Count ratio)=0.83	
Not already trying	Non drinkers: 38/78 (48.72%)<br>Mean in drinkers: 34.75 (44.77)	8/85 (9.40%)<br>30.18 (25.89)	aOR=8.30 (3.02, 22.75)<br>Count ratio=1.08 (0.66, 1.76)	P=0.001<br>P=0.48
Already trying	Non drinkers: 30/86 (34.88%)<br>Mean in drinkers: 38.61 (44.14)	23/87 (26.44%)<br>32.09 (30.05)	aOR=1.43 (0.17, 13.05)<br>Count ratio=1.07 (0.36, 3.11)	P=0.26<br>P=0.84
**Expectations of usefulness of counselling**			**Overall p-effect modification=0.97**[2]<br>p-effect modification (aOR)=0.96;<br>p-effect modification (Count ratio)=0.80	
Not or somewhat useful	Non drinkers: 12/36 (33.33%)<br>Mean in drinkers: 43.33 (35.96)	5/38 (13.16%)<br>37.21 (32.01)	aOR=3.65 (2.08, 6.42)<br>Count ratio=1.05 (0.80, 1.39)	P<0.0001<br>P=0.72
Moderately or very useful	Non drinkers: 56/128 (43.75%)<br>Mean in drinkers: 34.89 (46.67)	26/134 (19.40%)<br>29.17 (26.22)	aOR=3.05 (1.73, 2.08, 5.36)<br>Count ratio=1.01 (0.77, 1.34)	P=0.03<br>P=0.93

[1] Adjusting for counsellor clustering with robust standard errors
[2] Based on complete case analysis

Web-table 1: Comparison of participants who were followed up and LTFU at 3 months

	**Lost before 3 month evaluation (n=41; 11%)**	**Completed 3 month outcome evaluation (n=336; 89%)**	**p-value**
Age (years) (mean [SD])	38.5 (12.7)	42.4 (11.2)	0.04
**Marital status** (n [%])			0.07
Married	28 (68.3%)	273 (81.3%)	
Single	12 (29.3%)	58 (17.3%)	
Separated/Divorced	1 (2.4%)	1 (0.3%)	
Widowed	0 (0%)	4 (1.2%)	
**Occupation** (n [%])			0.20
Unemployed	2 (4.9%)	51 (15.2%)	
Unskilled manual labour	34 (82.9%)	232 (69.1%)	
Skilled manual labour	3 (7.3%)	22 (6.6%)	
Clerical & professional	2 (4.9%)	31 (9.2%)	
**Patient's expectation of counselling** (n [%])			
No/a little/somewhat useful	4 (9.8%)	74 (22.0%)	0.07
Moderately or very useful	37 (90.2%)	262 (77.8%)	
**AUDIT score** (median [IQR])	14 (13-16)	15 (13-17)	0.34
**AUDIT score** (mean (SD))	14.6 (1.9)	14.9 (2.1)	0.32
**AUDIT category** (n [%])			0.17
Score 12-15	30 (73.2%)	207 (61.6%)	
Score 16-19	11 (26.8%)	129 (38.4%)	

Web-Table 2: Serious adverse events by trial arm

Type of SAE	CAP Number of participants	EUC Number of participants	p-value
Any SAE	6	13	0.11
Death	0	3 [1]	0.25
Suicide attempt	0	3	0.25
Unplanned hospitalisation	6[2]	7	1.00

[1]One death from jaundice and kidney failure, one snake bite, one heart attack
[2]Two participants reported two unplanned hospitalisations each.

Web Table 3: Distribution of participation in the RCT by PHC

PHC	Total<br>n=678<br>n (%)	Consent to participate in the RCT		p-value
		No<br>n=301 (44.4)<br>n (%)	Yes<br>n=377 (55.6)<br>n (%)	
Bicholim	97 (14.3)	35 (36.1)	62 (63.9)	p=0.001
Candolim	81 (11.9)	43 (53.1)	38 (46.9)	
Siolim	51 (7.5)	27 (52.9)	24 (47.1)	
Sanquelim	111 (16.4)	51 (46.0)	60 (54.1)	
Valpoi	79 (11.7)	23 (29.1)	56 (70.9)	
Pernem	56 (8.3)	28 (50.0)	28 (50.0)	
Aldona	82 (12.1)	50 (61.0)	32 (39.0)	
Casarvarnem	88 (13.0)	29 (33.0)	59 (67.1)	
Betki	22 (3.2)	9 (40.9)	13 (59.1)	
Corlim/Divar	11 (1.6)	6 (54.6)	5 (45.5)	

Supplementary Figure1: Cost-effectiveness acceptability curve: willingness to pay per additional non-drinker achieved from Counselling for Alcohol Problems from a health system perspective.

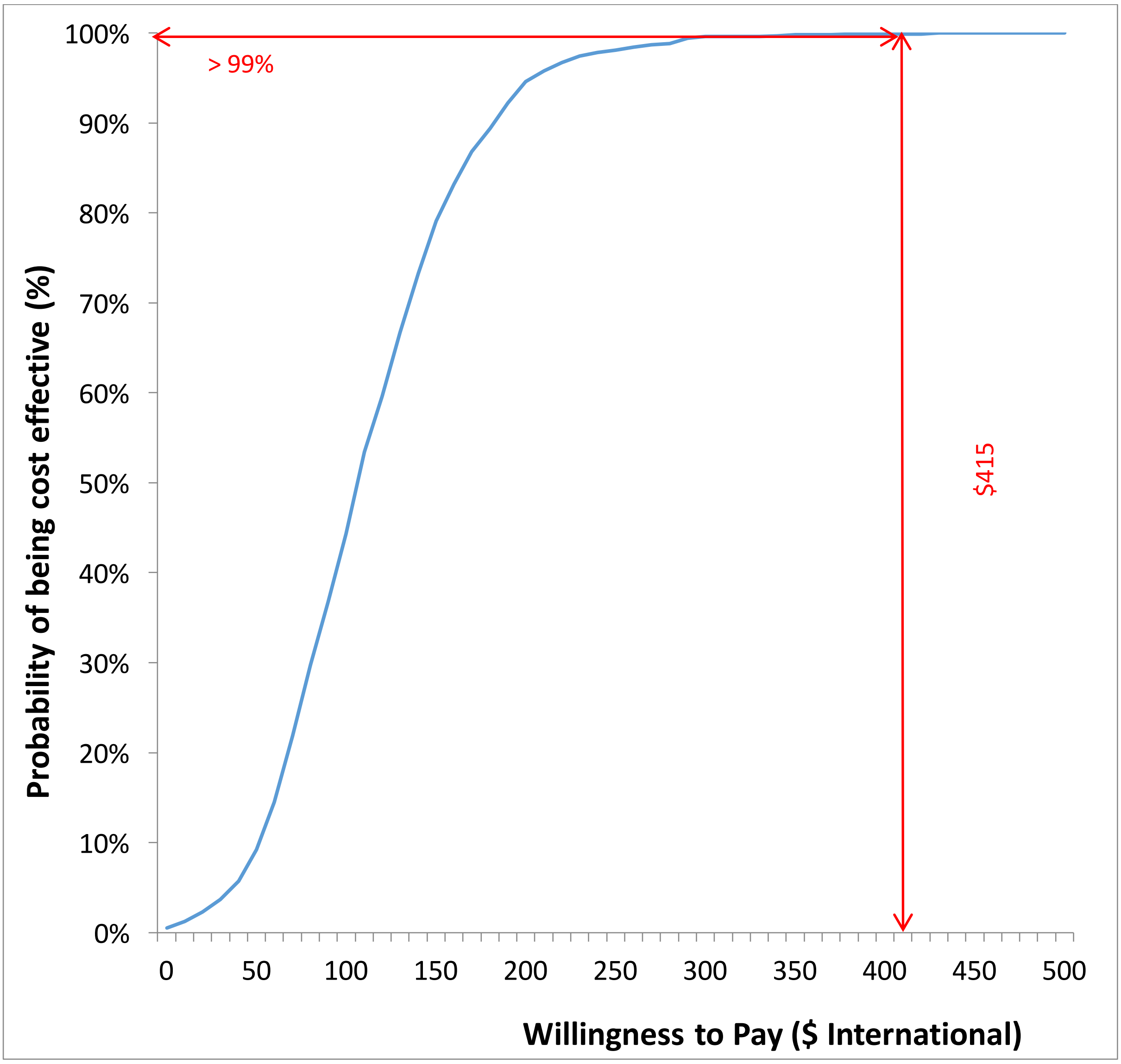

Supplement: Supplementary appendix [file mmc1.pdf]
